# The “Breastmilk Ecology: Genesis of Infant Nutrition (BEGIN)” Project – executive summary

**DOI:** 10.1016/j.ajcnut.2022.12.020

**Published:** 2023-05-10

**Authors:** Daniel J. Raiten, Alison L. Steiber, Constantina Papoutsakis, Mary Rozga, Deepa Handu, Gabriela V. Proaño, Lisa Moloney, Andrew A. Bremer

**Affiliations:** 1 Pediatric Growth and Nutrition Branch, *Eunice Kennedy Shriver* National Institute of Child Health and Human Development, National Institutes of Health, Bethesda, MD, USA; 2 Academy of Nutrition and Dietetics, Chicago, IL, USA

**Keywords:** human milk, infant feeding practices, breastfeeding, breastfeeding parent–infant dyad

## Abstract

The public health community has come to appreciate that a deeper understanding of the biology of human milk is essential to address ongoing and emerging questions about infant feeding practices. The critical pieces of that understanding are that *1*) human milk is a complex biological system, a matrix of many interacting parts that is more than the sum of those parts, and *2*) human milk production needs to be studied as an ecology that consists of inputs from the lactating parent, their breastfed baby, and their respective environments. The “Breastmilk Ecology: Genesis of Infant Nutrition (BEGIN)” Project was designed to examine this ecology as well as its functional implications for both the parent and infant and to explore ways in which this emerging knowledge can be expanded via a targeted research agenda and translated to support the community’s efforts to ensure safe, efficacious, and context-specific infant feeding practices in the United States and globally. The five working groups of the BEGIN Project addressed the following themes: *1*) parental inputs to human milk production and composition; *2*) the components of human milk and the interactions of those components within this complex biological system; *3*) infant inputs to the matrix, emphasizing the bidirectional relationships associated with the breastfeeding dyad; *4*) the application of existing and new technologies and methodologies to study human milk as a complex biological system; and *5*) approaches to translation and implementation of new knowledge to support safe and efficacious infant feeding practices.

## Introduction

Human milk has proven to be the ideal personalized food to support the health and development of infants (see Text Box 1 for core concepts and terms related to the project described herein). Awareness of the public health importance of human milk in the health and development of infants has been shown through studies demonstrating the impact of infant feeding practices on short- and long-term health outcomes. The importance of human milk was reinforced by efforts beginning in 2014 with the initiation of the “Evaluating the evidence base to support the inclusion of infants and children from birth to 24 mo of age in the Dietary Guidelines for Americans—the B-24 Project” [1], and ultimately codified with the inclusion of dietary guidelines for pregnant and lactating parents and children from birth to 2 y of age in the 2020-2025 U.S. Dietary Guidelines for Americans (DGA) [2]. Comprehensive systematic reviews were conducted and used as the foundation for the DGA. These highlighted both the existing evidence for the importance of diet in these critical periods of human development and the key research gaps and data needs to support current and future efforts [3].

Text Box 1 **Core concepts and terms.**

• In the context of this paper, “ecology” is defined as a complex biological system and its interactions with its environment. In this case, the complex system is human milk composition and its inherent biology and the environment consists of parental and infant inputs and the influence of their respective internal and external environments.
• With due recognition of the need to be observant of issues of gender identity or neutrality and to improve precision, to the extent possible, for the purposes of the papers described herein, we will use gender-neutral terminology where appropriate (e.g., lactating parent or person etc.) to reflect the reality that not all who lactate identify as female. The term “lactating parent” respects and recognizes those who may have been born female but do not identify as such and other gender-relevant contingencies. In situations where reporting primary data (studies or analyses), we will refer to the population as specified (e.g., “the study evaluated 250 lactating mothers”). Moreover, rather than using terms such as “maternal” or “maternal milk,” we will use the terms such as “birthing parent” throughout the report as appropriate as they accurately reflect the biological nature of the birthing parent–infant dyad.
• “Human milk” refers to milk produced by lactating parents and includes both *1*) breastmilk produced by a parent for their infant and fed directly to infants via the breast or expressed by the lactating parent and then fed to the infant and *2*) donor or banked human milk produced by lactating persons that is either donated to human milk banks or fed to infants other than their own child.
• “Nutrients” refer to dietary essential macronutrients (protein, fat, carbohydrates, and subcomponents [e.g., amino and fatty acids]) and micronutrients (vitamins and minerals).
• “Bioactive components” (nonnutritive) refer to those endogenously produced components in human milk that have been identified and found to exert a biological effect either within the matrix or in the context of parental or infant health. Exogenously produced compounds (e.g., toxins and drugs) are broadly classified as “xenobiotics.”

Alt-text: Text Box 1

To support public health goals, infant nutritional requirements must be better defined. As such, it is critical to understand the biology of lactation and human milk. This understanding will not only support breastfeeding but also inform efforts to ensure nutritional adequacy of both term and preterm infants who, for various reasons, might not have access to their lactating parent’s own milk (POM). The ability to provide safe and nutritious alternatives to POM—such as banked human milk or infant formulas—demands a comprehensive understanding of the composition of human milk throughout normal lactation and of, as in the case of prematurity, what happens when that process is disrupted/interrupted. The issues pertaining to the nutritional needs of preterm infants have been recently highlighted in the Pre-B Project [4], which was similar in design and approach to the initial B-24 Project and highlighted key research and data needs.

These efforts have reinforced our understanding of human milk as a source of several key nutrients and bioactive elements needed by the infant, but these efforts also reveal a lack of understanding about the ontogeny of human milk and the critical nature of the infant feeding “triad” (i.e., the intersection of parental and infant inputs and the milk matrix, including the nature of the relationships between the myriad components of human milk). Given the numerous factors that go into infant feeding choices, how do we use this new knowledge to support safe and efficacious infant feeding practices?

Despite the strong communal commitment to support breastfeeding, many questions remain, and efforts to develop public health guidance continue to be thwarted by a lack of evidence—exemplified by the following statement by the recent DGA Committee: “Despite the importance of the topics examined for the long-term health of the child, the available evidence for many questions was insufficient to form conclusion statements, highlighting the critical need for additional research” [2].

This lack of evidence was further reinforced by the recent report from the National Academies of Sciences, Engineering, and Medicine (NASEM) Committee on Scanning for New Evidence on the Nutrient Content of human milk [5]. The goal of the NASEM report was to determine the extent and quality of evidence from studies of human milk composition that could be usefully applied to the development of DRI for infants’ aged 0–2 y. Text Box 2 is a summary of some of the key points of the exercise [5].

Text Box 2 **Summary of NASEM report on human milk composition and volume [5].**

Data:

• Initial search resulted in 42,762 relevant papers on human milk composition and volume.
• 1901 articles remained after the removal of duplicates and title and abstract screening.
• 126 articles remained for inclusion after full-text screening and subsequent data abstraction.

Results:

• “The selected studies represent the committee’s assessment of data that would provide the most reliable estimates of human milk composition and volume.”
• The committee highlighted several inconsistencies in study designs as follows:
  o Inconsistency in describing study’s subjects as a healthy population.
  o Variability in the quality of methods used to analyze the milk composition.
  o A lack of data reported for fluoride; biotin; molybdenum; niacin; pantothenic acid; riboflavin; thiamin; and vitamins B_12_, C, D, and K.

Conclusions:

• “… for the purposes of informing the development of future DRI values for infants from birth to 12 mo of age, the committee found that although it is possible that adequate numbers of participants have been studied so that new DRI values could be developed from the currently available data for some nutrients, for other nutrients, additional research will be needed.”
• “Other shortfalls noted by the committee included consistency in milk sampling protocols and a definition of an appropriate milk sampling strategy.”
• “Lastly, the committee recognized the importance of continuing research into less well-understood milk components (e.g., oligosaccharides, bioactive substances, food antigens, hormones, microbes, and growth factors) that contribute to infant growth, development, and overall health.”

Alt-text: Text Box 2

Thus, the two most recent and comprehensive attempts at identifying and utilizing the extant data on human milk composition to address critical issues in public health nutrition and infant feeding practices clearly highlight *1*) the lack of available high-quality data generated from consistent and reproducible experimental design or methodologies and *2*) an ongoing focus mainly on only one of the myriad aspects regarding the biology of human milk (i.e., individual components).

To move forward, we need a knowledge base that can inform relevant evidence-based standards to support safe and efficacious infant feeding practices and nutritional care for infants, particularly those with special needs such as preterm infants. Similarly, new knowledge is needed to support agencies charged with adjudicating the safety of human milk sources other than POM (e.g., banked/donor human milk) and the safety and efficacy of ingredients proposed for addition to breastmilk substitutes (e.g., infant formula).

Core questions to be addressed to support this agenda might include:

• How do we understand the biological inputs of the lactating parent to the production and composition of human milk?
• How do we study and understand human milk as a biological system (e.g., an ecology) consisting of multiple interacting components existing within a matrix that is more than just the sum of its individual parts?
• What is the role of infant factors and the dyadic interaction between the infant and lactating parent in influencing lactation and human milk composition?
• How can we exploit the existing and emerging new technologies in the design of new research to understand this complex system and its ecology?
• How can we best translate our understanding of human milk biology to improved infant feeding practices and improved parental care?

Although much is known about human lactation, much remains to be learned as there are significant gaps in our understanding of the developmental biology of the mammary gland, the ontogeny of human milk, and the biological and social, cultural, or environmental factors that can impact a parent’s ability to successfully breastfeed. New information has also emerged on the potential impact of additional factors such as parental body composition, the presence of infectious/noncommunicable diseases or inflammation, parental and/or infant exposure to xenobiotics including pharmacotherapeutics, infant factors, genetic or epigenetic conditions, and the effects of the parental and infant microbiomes on successful implementation and continuation of breastfeeding. Significant gaps also exist regarding the nutritional needs of lactating parents to support breastfeeding, human milk production or composition, and parental health. Finally, there are significant gaps in evidence to inform the composition of infant formulas to achieve the same physiological effects as human milk. The ability to address these issues will provide us with a better opportunity to address some of the key questions facing the infant feeding enterprise such as those highlighted in Text Box 3.

Text Box 3 **Key questions that might be addressed by improved understanding of human milk biology.**

• What is the role of genetics, health, and environmental exposure in the development and function of the mammary gland, and what are the implications of those factors for both lactation and human milk composition?
• What are the precise nutritional requirements and optimal feeding practices for term or preterm infants and their lactating parents?
• How can we best develop and assess standards for lactation initiation and sustainability with due consideration of issues such as context, equity, and chronobiology of human milk production?
• How can an expanded understanding of the biology of human milk and its components be used to inform practice-based recommendations for the following:
  o Duration of exclusive breastfeeding
  o Timing and use of expressed human milk
  o Value of donor or banked human milk
  o Timing and composition of complementary foods
• If human milk is an active, interacting biological system, what is the role and impact of the human milk matrix?
• How can a deeper understanding of human milk composition and biology inform infant feeding practices in the context of infectious and noncommunicable diseases and their treatment?
• How can the emerging knowledge of human milk composition and biology inform the evaluation of the safety and efficacy of human milk substitutes?

Alt-text: Text Box 3

## The BEGIN Project

The *Eunice Kennedy Shriver* National Institute of Child Health and Human Development (NICHD) of the National Institutes of Health (NIH) has historically played an integral role in developing the evidence base to *1*) improve the survival of infants born preterm or at low birthweight and *2*) optimize the outcomes of all infants by understanding the roles of early interventions, nutritional factors, the microbiome, and social and environmental determinants of health. Of unique interest to the NICHD is a better understanding of the composition and function of human milk [6], the effect of parental nutrition and length of gestation on human milk composition and lactation, and the optimal source of nutrition and mode of nutrient delivery to the infant, including preterm infants.

To address these and other mission-relevant issues, NICHD initiated the “Breastmilk Ecology: Genesis of Infant Nutrition (BEGIN)” Project in 2020. The overarching objective of the BEGIN Project is to explore factors influencing the synthesis, composition, and best use of human milk. The BEGIN Project represents a paradigm shift away from the focus solely on human milk as a delivery system for individual components to a more expansive view of human milk as a complex biological system influenced by various environmental inputs (i.e., an ecology). Our ability to answer current and emerging questions about that ecology will inform efforts to support context-specific, equitable, safe, and efficacious infant feeding practices in the United States and globally.

### Purpose

The goal of the BEGIN Project is to support efforts to generate evidence to answer key questions about human milk as a unique biological system and its intersection with both the internal (biology) and external (physical, social, or behavioral) ecologies. Importantly, the goal is not to generate programs, policies, or standards of care or to advocate for any aspect of infant feeding. Rather, our focus is to stimulate the generation of evidence needed to support those agencies, organizations, and stakeholder groups in the public and private sectors that are involved in these aspects of infant feeding care.

### BEGIN structure and process

To achieve the Project’s goals, the BEGIN process included the following components:

• BEGIN Secretariat: consisted of the program staff from NICHD and members of a collaborating research team from the Academy for Nutrition and Dietetics (Academy).
• Scientific Steering Committee (SSC): consisting of representatives from several United States Government agencies and professional organizations involved in various aspects of the infant feeding or public health enterprise (see Supplemental Table 1 for listing of SSC members).
• Thematic working groups (WGs): (see Supplemental Table 2 for WG members).

### Charge of the WGs

The WGs were charged with developing narrative reviews of the extant literature for their respective themes. The goal was not to develop guidelines, standards of care, or dietary recommendations. Rather, the WGs were each asked to conceptualize a new approach to the understanding of the role of their respective components of the infant feeding triad and its implications for human milk composition and infant feeding practices.

### Process

After a series of conference calls, each WG had the opportunity to convene virtual workshops that were designed to *1*) support their efforts to develop outlines and approaches to their reports, *2*) invite additional expertise to support their efforts, and *3*) have an opportunity to inform and be informed by the other WGs. The WGs were encouraged to work together wherever possible to ensure comprehensive coverage of the most relevant issues, avoid redundancy, and ensure a coordinated flow within and across each of these themes.

The following is a brief summary of the content and, more importantly, the operating principles adopted by each WG in their efforts to address the core questions listed above.

### WG 1: Parental Factors that Impact the Ecology of Human Mammary Gland Development, Milk Secretion, and Milk Composition

#### Organizing principles

To explore what is known and not known about *1*) the role of the lactating parent and the development of human milk and *2*) critical factors that influence human milk development in the context of infant and parental health and disease [7]. In addition to these core questions, WG 1 addressed the following:

• How does mammary gland development, particularly during pregnancy, affect the ability of the gland to secrete milk?
• What is the input of the infant to the process of secretory activation and milk secretion?
• How do disease conditions, inflammation, preterm birth, and, in particular, birthing parent’s obesity impact the process?

WG 1 also considered many of the inadequately understood factors that impact milk volume and composition in the lactating parent including those highlighted in Text Box 4 [7].

Text Box 4 **Specific issues covered by WG 1.**

• Breast anatomy
• The vascular system
• The endocrinology of lactation
• Diet or nutrition
• Chronobiology: how both the time of day and postpartum interval impact milk synthesis
• Understanding the role and mechanisms of parent–infant interactions in milk secretion and bonding from the parental perspective
• The impact of parental health on breast development, including the effects of preterm birth, cardiovascular health, inflammatory states, and particularly gestational diabetes and obesity
• Impact of parental xenobiotics (i.e., environmental agents including medications, recreational and illicit drugs, pesticides, and endocrine-disrupting chemicals) on mammary gland development, function, milk secretion, and composition
• The immunology of lactation (i.e., the integration of immune components of human milk and relevant aspects of the parental response to infection)

Alt-text: Text Box 4

In addition to its unique perspectives on those factors impacting parental inputs to lactation and human milk composition, WG 1 offered a series of targeted research suggestions.

### WG 2: Ecologies, Synergies, and Biological Systems Shaping Human Milk Composition

#### Organizing principles

To *1*) categorize the components of human milk, recognizing that these components can perform several functions broadly defined as nurturing, protecting, and communicating; *2*) characterize human milk as a biological system; and *3*) elucidate how these functions and interactions have implications for both the infant and the matrix [8].

WG 2 endeavored to develop a conceptual framework that would represent both the roles and functions of the specific components of human milk and a deeper appreciation of human milk as a biological system, with a particular focus on the nature and function of the human milk matrix as a whole and the interactions of the components within that matrix. In addition to recognizing the complex functions of human milk components, WG 2 also explored how that matrix might impact infant health.

The unique perspectives presented by WG 2 of what human milk components do and how they interact within the human milk matrix present an important expansion from the traditional view of human milk as simply a source of these components for the infant. While the latter remains an important function of human milk, this new perspective offers a deeper appreciation of this system that will allow us to better explore how those factors within both the parent’s and infant’s environments explored by WGs 1 and 3 affect these fundamental and critical relationships. Importantly, with this new perspective, WG 2 challenges us to begin thinking more critically about how best to study human milk, its components, the human milk matrix, and their respective impacts on parental and infant health.

### WG 3: Infant Factors that Impact the Ecology of Human Milk Secretion and Composition

#### Organizing principle

To address how the normal functional relationships between the infant and human milk composition, secretion, and release are impacted by both lactating parent and infant health and environmental exposures (including therapeutic and recreational drugs) [9].

In many respects, WG 3 adopted a similar approach as WG 1 (i.e., asking the question of what is “normal” and what is “not normal” regarding the nature of the infant and human milk interactions and, more expansively, the infant–parent dyadic relationships).

A critical element of the WG 3 report is the coverage of several key crosscutting issues that describe the intimate and intersecting ecologies of both the lactating parent and the infant. Building on the reports by WGs 1 and 2, these issues include those highlighted in Text Box 5 [9].

Text Box 5 **Specific issues covered by WG 3.**

• Both pre- and postnatal parent–infant interactions affecting the human milk microbiome.
• Xenobiotics (with particular reference to the implications of recreational drugs) and their impact on infant health and feeding behaviors.
• The factors predicting and affecting prematurity and infant feeding practices (e.g., intrauterine environment, birthing parent obesity, or the presence of noncommunicable diseases).
• Extension of the coverage of WG 1 of the endocrine or paracrine system and the parental regulation of human milk production with a review of the factors contributing to milk removal from the infant’s perspective and the implications of those processes for human milk biology and composition.
• Sensory development of the infant and the interplay of that development with the parental environment (e.g., the intimate and inextricable relationships between parental dietary exposures during pregnancy and lactation and their impact on the entrainment of the infant’s sensory responses and the reciprocal impacts on breastfeeding behavior and biology)

Alt-text: Text Box 5

### WG 4: Evidence for Human Milk as a Biological System and Recommendations on Study Design

#### Organizing principle

To address how human milk is a unique biological system that will demand new and creative approaches in basic, clinical, translational, and population-based research [10]. This new perspective will demand a multidisciplinary approach that will engage state-of-the-art technologies in systems biology, relevant “-omics,” and integrated approaches to big data.

Based on the critical aspects of the human milk ecology from both the parental and infant perspectives along with the strong case for viewing human milk as a unique biological system meticulously outlined by WGs 1-3, the essential challenge moving forward will be how to utilize existing and emerging methodologies in basic, clinical, and translational science to validate this conceptual framework.

WG 4 covered this challenge from three perspectives and used evidence to support them, including:

• An examination of the methodological and/or design limitations of current evidence for human milk as a biological system affecting development and clinical or functional outcomes
• A review of information on the functionality of components of human milk, alone or in combination, and the impact on infant development
• Presentation of suggestions for the experimental framework and analytical approaches needed to evaluate the functional implications for the lactating parent and infant of the full range of factors comprising the internal and external human milk ecology, including the role of human milk components and the human milk matrix

### WG 5: An Equitable, Community-Engaged Translational Framework for Science in Human Lactation and Infant Feeding

#### Organizing principle

To address the need for a framework to describe the process of translating new human milk and lactation evidence to improved guidance, standards of care, and policy [11]. This framework must reflect the need for an equitable, nonlinear process that integrates the full continuum of efforts from basic, clinical, translational, and epidemiological research to implementation science; integrates those environmental factors (i.e., physical, social or behavioral, demographic, and structural) that impact message development and adaptation; and support context-specific programs, policies, and interventions.

The components of the framework suggested by WG 5 are outlined in Text Box 6 [11].

Text Box 6 **Proposed framework for translational themes (T).**

T1—Discovery

• Objective: to address the fundamental questions of observing, identifying, and understanding human lactation and infant nutrition at the discovery level.
• Approach: cell models and other basic sciences; animal models of lactation and infant nutrition; and observational studies in human cohorts, especially lactating parent-infant cohorts.

T2—Human health implications

• Objective: to apply discoveries toward improving the understanding of health implications in lactating parents and human milk–fed infants and children.
• Approach: highly structured, focused human experiments to establish causation, assess feasibility, develop methods for assessment, or validate prediction tools.

T3—Clinical and public health implications

• Objective: to utilize the smaller and highly focused work of T2 research to progress to large-scale testing of a validated approach to improving an outcome.
• Approach: intervention studies aimed at testing a hypothesis established from T2 research to determine clinical or public health implications in real-world settings. Scaled up intervention studies may include individually or cluster-randomized controlled trials, randomized crossover trials, patient-centered outcomes research, and comparative effectiveness trials.

T4—Implementation

• Objective: to inform, develop, or implement evidence-based protocols, guidelines, or policies for implementation in clinical, public health, or community settings.
• Approach: systematic reviews and meta-analyses, risk communication research, shared decision-making research, and implementation research.

T5—Impact

• Objective: to utilize surveillance mechanisms to assess attainment of health goals within the healthcare system, community, nation, region, or globally.
• Approach: utilization, expansion, or de novo development of surveillance infrastructure to assess the outcomes of relevant interventions and evaluate unintended consequences and demographic disparities in meeting goals.

Alt-text: Text Box 6

Conceptually, the stages of this proposed framework overlay with the NIH’s mission, which is “…to seek fundamental knowledge about the nature and behavior of living systems [T1, T2] and the application of that knowledge [T2, T3] to enhance health [T3, T4], lengthen life [T5], and reduce the burdens of illness and disability [T5].”

WG 5 applied this framework to six case studies focused on issues of particular public health interest:

• Development of dietary guidelines for lactating parents
• Insufficient milk production in the context of obesity
• Cannabis use during lactation
• Duration of exclusive breastfeeding
• Individualized fortification of human milk for preterm infants
• Optimizing the provision of human milk in low- and middle-income countries (LMIC)

## The Value of an Application of the BEGIN Ecological Approach: Case Studies

The breastmilk ecology is complex, and the WG reports highlight the contributions of the key elements of the ecology from the perspective of the infant feeding “triad” (i.e., parent, human milk matrix, and infant). In addition to the focus on the biology covered by WGs 1–4 [[7], [8], [9], [10]], WG 5 [11] applied an innovative framework for translating the emerging knowledge to meaningful messaging/policies and context-specific interventions. The intention of BEGIN was to highlight the need for this more comprehensive view of human milk biology and how that perspective may be applied to advancing our understanding of the clinical and public health implications of these complex interactions. The following examples are intended to highlight the need for and value of the application of an ecological approach to a set of cross-cutting issues.

### Case study 1: the interacting microbiomes

WGs 1–3 [[7], [8], [9]] covered various aspects of those parental and infant factors that impact the human milk microbiome and its reciprocal impact on both the lactating parent and the infant. Table 1 highlights some of the key points of WGs 1–3. Clearly, our ability to understand the complex nature of these relationships and apply that understanding to the development of evidence-based programs/policies and guidance demand a comprehensive and multidisciplinary approach such as that highlighted by WGs 4 and 5 [10, 11].

**TABLE 1:** WG perspectives on the interacting microbiomes

WG 1	WG 2	WG 3
• Factors affecting parental transmission of microbes to human milk • Internal parental ecology (i.e., the intersection of the parental “gut/mammary axis”) • Role of parental diet and nutrition including the use of probiotics • Genetic and dietary influence on the production of human milk oligosaccharides and subsequent impact on infant microbiome and gut development	• Microbial composition of human milk • Roles of the human milk microbiome within the context of the “nourish, communicate, and protect” paradigm • Interactions among components within the human milk matrix (e.g., macro- or micronutrients, HMOs, and the constituents of the human milk microbiome) • Potential impacts of environmental toxins on the human milk microbiome	• Parental inputs • Timing and development of infant oral or gut microbiome • Impact of the feeding dyad on human milk microbiome • Intersection of human milk components and the development, composition, and stability of the infant microbiome

HMO, human milk oligosaccharide; WG, working group.

Text Box 7 highlights just a few of the kinds of questions that have surfaced with specific regard to the human milk microbiome and its intersection with the parental and infant ecologies. The ability to address these or other questions related to this important feature of human milk biology demands a comprehensive approach that recognizes the importance of an application of this ecological complex systems approach.

Text Box 7 **Application: research questions—parent–human milk–infant microbiomes.**

• How does the parental microbiomes (gut, vaginal, and oral) impact the composition of human milk and subsequent implications for their breastfeeding infant?
• How are those parental microbiomes affected by:
  o Genetics?
  o Disease (infectious or noninfectious)?
  o Environmental exposures (including xenobiotics)?
  o Diet?
  o Nutritional status?
• Is there a chronobiology to the parental microbiomes?
  o Does the human milk microbiome change over time, and what might be the implications of those differences to the infant?
• Are there differences in exposure scenarios between infants receiving expressed milk and those who are breastfed?
• Is there a role for pre- or probiotics to manipulate the parental microbiome? If so, what would be the impact on human milk and subsequent implications for the breastfed infant?
  o Are there differences to be considered between pre- and probiotics for the lactating parent vs. the infant?

Alt-text: Text Box 7

### Case study 2: xenobiotics

We live in a complex environment consisting of numerous exposures to various chemical entities from toxins to therapeutics, which, increasingly, also includes various recreational drugs and a myriad of nonnutritional bioactive dietary supplements. How the lactating parent’s inherent biology responds to these exposures and their implications for their health, the composition of their milk, and ultimately the health of their infant is a compelling public health concern. Again, it is not simply a matter of addressing one aspect of this complex ecology, but rather the need to systematically explore the critical elements of these potential interactions within the infant feeding triad to provide accessible, valid, and useful messages and standards of care to providers and patients alike (Table 2).

**TABLE 2:** WG perspectives on xenobiotics

WG 1	WG 2	WG 3
• There is a considerable lack of understanding concerning the effects of xenobiotics on lactational physiology, lactational neuroendocrinology, and the behavioral component of parent–infant bonding • Parental pain, anger, confusion, and/or frustration are associated with a reduction in the normal pulsatile frequency of oxytocin and a 30%–50% reduction of milk volume per nursing episode • The major neurotransmitters affecting lactation include dopamine D2, serotonin 5-HT2A, and/or serotonin 5-HT1A receptors • Common psychotropic medications affect these neurotransmitter pathways and have been shown to impact the triad including: o Antipsychotics (e.g., phenothiazines that target the neurohormones associated with oxytocinergic pathways) o Antidepressants (e.g., serotonin receptor uptake inhibitors [SSRIs] that delay secretory activation in lactating parents) o Recommendations for the use of these drugs in breastfeeding parents have not been formulated • Recreational drugs (e.g., tobacco, alcohol, cannabis) o Tetrahydrocannabinol (THC) and cannabidiol accumulate at significant levels in breastmilk relative to plasma; however, their effects on lactation and the recipient infant are unknown • Environmental toxins (e.g., endocrine disrupting chemicals [EDCs]) o Exposure to EDCs is associated with the dysfunction of hormone-mediated processes such as metabolism, energy balance, thyroid and reproductive functions, immune functions, and neurodevelopment	• A myriad of bioactive components of plant- or animal-source foods have been found in human milk. While the potential impact of some of these compounds have been studied in parents and infants, their implications for the biology of human milk have not been studied • Numerous environmental toxins have also been found in human milk. While their potential impact on human health has been examined, their implications for human milk biology have not been examined	• Alcohol and the active ingredients in tobacco (nicotine) and cannabis (THC) are found after parental consumption in human milk and are transferred to infants • The presence of these substances may affect infant’s taste preferences and may have long-term implications for feeding behavior • While there is evidence of the impact of these exposures on infant feeding, sleep, and other behaviors, it is difficult to determine the direct cause and effect due to the confounding impacts of drug exposure on parental neuroendocrinology and behavior

WG, working group.

There is no question that human milk is a destination for an array of food and environmental bioactive compounds including toxins, drugs, and their metabolites. What is less understood is the biological significance of their presence, particularly regarding the biology and ecology of human milk. The coverage of WGs 1–3 [[7], [8], [9]] reinforces both the complexity of the intersection of xenobiotics, human milk components, and infant feeding. This complexity is exemplified by the critical challenge of how to design research that will be able to untangle the impact of parental behavior (predisposition and response) and infant’s response to xenobiotics whether used therapeutically or “recreationally.” Given the prevalence of the use of these substances and the increasing exposure to all manner of environmental toxins, this is an area of growing importance. Finally, given the importance of sociodemographic and related factors affecting the prevalence and context of exposure, the elements outlined in the Translational Framework of WG 5 [11] will assume a major role in the design of the research, as well as the development and implementation of context-specific programs, guidance, and standards of care. Some research priorities are highlighted in Text Box 8.

Text Box 8 **Research questions—human milk and xenobiotics.**

• What is the prevalence of exposure in lactating parents to xenobiotics including:
  o Food-derived bioactive compounds?
  o Therapeutic drugs (chemotherapeutics, psychotropics [including antipsychotics and antidepressants], etc.)?
  o Recreational drugs (e.g., cannabis, opioids, alcohol, and tobacco)?
  o Environmental toxins (e.g., endocrine disruptors)?
• Do xenobiotics alter the physiology of milk production or secretion and its quality?
• Do medications, especially psychoactive medications, affect oxytocin or prolactin release resulting from the stimulation of the nipple by the breastfeeding infant?
• Do medications (i.e., psychoactive medications such as antidepressants, antipsychotics, hypnotics, antihistamines, and narcotics) affect parent–infant bonding?
• Do cannabinoids or heavy alcohol use affect:
  o The let-down reflex?
  o Mother–infant bonding and the response to stress?
• How do low doses of endocrine disrupting chemicals and their metabolites affect the neuroendocrinology of lactation?
• Do xenobiotics and their metabolites assume some role within the human milk matrix within the protection or communication paradigm?
• What are the impacts of xenobiotics on the biology of the human milk matrix?
• What are the potential long-term impacts of exposure to recreational drugs (e.g., cannabis, alcohol, and tobacco) via human milk on infant behavior, growth, and neurological development?
• What are effective, nonstigmatizing strategies for motivating parents to reduce recreational drug use without undermining breastfeeding?

Alt-text: Text Box 8

### Case study 3: infectious diseases

A critical challenge facing the global public health community is to determine whether or how to respond to infectious diseases and their potential impact on human milk biology and infants’ feeding practices. Over the past 20 y, we have been confronted with multiple infectious diseases that had a global impact. Each has caused an array of responses including confusion and fear with specific regard to how best to respond in the context of safe and efficacious infant feeding practices. Whether it is HIV-1, Ebola, or COVID-19, the spread of infectious diseases has highlighted the urgency of improving our approach to the research and understanding of the intersection of infection and all aspects of lactation and infant feeding practices. Each of the BEGIN WGs addressed this intersection in the context of the human milk ecology and in so doing reinforced the importance of a systems approach to fully address these challenges (Table 3).

**TABLE 3:** WG perspectives on infectious diseases

WG 1	WG 2	WG 3
• Only some infectious organisms have been reported to be transmitted and to be infectious via human milk • Bacteria: MTB and certain Streptococci o Transmission of both could be through lesions on the breast o Presence and transmissibility may be context-specific (e.g., geography) • Viruses shown to be transmitted in human milk include: *1*) HIV-1; *2*) CMV; *3*) HTLV-1; *4*) DENV; and *5*) ZIKV o Mechanism of secretion differs for most of these viruses o HIV-1 may be translocated across the mammary gland epithelium o The advent of highly active antiretroviral therapies (HAAARTs) has significantly reduced HIV transmission from the birthing parent to the child, presumably by decreasing the viral load in the lactating parent and human milk o HTLV is likely translocated across the mammary gland epithelium by leukocytes o CMV is reactivated in mammary gland cells and shed into the milk o The mechanisms by which DENV and ZIKV translocate to human milk are not yet known, but these viruses must have some way of crossing the mammary gland epithelium into human milk • To date, there is no evidence of transmissible, infective SARS-COV-2 in human milk	• Human milk also contains myriad individual biologically active proteins that play extensive roles in protection and communication • Secretory IGA (sIgA), the most abundant immunoglobulin in human milk, may provide unique vertical transmission of immune system education • sIgA in milk serves a dual function by protecting newborn infants from infection by neutralizing pathogenic bacteria and at the same time shaping the commensal GI microbial community structure • Specificity of sIgA in milk reflects prior parental exposures • sIgA has also been shown to affect host–microbiome interactions by modulating the colonic transit time of bound microbes and binding to antigens originating from the lactating parent’s diet • Leukocytes are the predominant cells in colostrum and early milk • Leukocytes are presumed to protect the mammary gland from infection during lactation but also may respond to the health of the infant as concentrations increase during parental or infant infection • In vitro studies suggest that the leukocytes present respond to viral antigens with increased proliferation rate and altered expression of IL-6, IL-17A, interferon-g, and tumor necrosis factor-a demonstrating potential roles to deliver protection in the form of innate immunity to the infant • There is evidence suggesting that several metabolically active components of human milk may be involved in interactions that may provide protection (e.g., xanthine oxidase reductase [XOR] enzymatic activity produces reactive oxygen and nitrogen species that have been shown to have antimicrobial and infant immune defense properties) • WG 2 provided several other examples of how the complex interactions of components within the milk matrix may confer protection	• Active infection in infants from a variety of ailments, including influenza, measles, and respiratory and gastrointestinal infections, has been associated with an increase in human milk leukocyte concentrations, which quickly return to baseline after recovery • The mechanisms for this response are unclear; however, it has been suggested that *1*) active infection in the infant either co-occurs with or results in an infection of the parent and/or *2*) the presence of infant infection signals the need for an inflammatory lactating parent response • Both mechanisms result in increased secretion of white blood cells and cytokines into milk, a cascade of events that could potentially have the dual effect of attacking infectious organisms while also adversely impacting the human milk microbiome • Antibiotic use by the lactating parent has been shown to change the human milk microbiome • Antibiotic use during infancy alters the gut microbiome of the infant. Whether an altered infant gut microbiome can have a clinically relevant impact on the human milk microbiome remains to be determined

CMV, cytomegalovirus; MTB, mycobacterium tuberculosis; SARS-COV-2, severe acute respiratory syndrome coronavirus 2

The key components of an approach to research to support resiliency in our response to these global challenges are highlighted in Text Box 9.

Text Box 9 **Application: research questions—human milk biology and infectious diseases.**

• Does the infectious organism enter human milk?
• Is it transmissible to the infant?
• Does it change the immune components of human milk?
• Does it change the composition of other nutritional or bioactive components of human milk?
• If so, do these changes have functional implications for the infant?
• What are the impacts of therapeutics in this context on:
  o Parental health and behavior relevant to infant’s feeding practices?
  o Human milk composition and biology?
• Does the infection affect health and nutrition of the lactating parent in a manner that has implications for human milk composition and/or lactation biology?
• Are there differences between infants exposed but uninfected and those who are exposed and infected in terms of:
  o Feeding behavior?
  o Growth and development?
  o Nutritional requirements?
  o Long-term health outcomes?
• What are the considerations in the development of context-specific strategies for communicating the benefits of breastfeeding and risk of infectious disease transmission?

Alt-text: Text Box 9

## Conclusions

The three case studies mentioned above are a small reflection of the kinds of questions facing the lactation and infant feeding community. The BEGIN Project was designed to start a process by which our understanding of human milk can evolve beyond the traditional focus on individual constituents of human milk. The results of the WG deliberations are reflected in the five papers comprising this supplement. What is clear from the WG reports is that we have an evolving knowledge base to begin to explain:

(1) The mechanisms of human milk formation and factors affecting the lactating parent’s relevant biology.
(2) Human milk composition in terms of both nutrients and bioactive components and the potential role of the human milk matrix as a functioning biological system.
(3) The relative impacts of both the parental biology and inputs from the infant on human milk.
(4) Finally, an emerging understanding of the implications of environmental exposures and adverse health for these processes.

In all cases, an exquisitely detailed research agenda has been presented to inform future work in these areas, including an exploration by WG 4 of ways by which this complex biological system may be explored using current and emerging approaches in systems biology. Finally, an overarching theme of the BEGIN process is the recognition of the need for a fuller appreciation of the continuum of effort needed to develop both new evidence and the translation of that evidence to context-specific, safe, and effective programs; policies; and interventions to support best practices in infant feeding. The translational framework developed by WG 5 not only provides a path forward but also highlights the need for authentic, equitable stakeholder engagement and a consideration of the context-specific issues confronting healthcare providers, parents, and caregivers. The framework emphasizes the need to be inclusive not only in terms of our research enterprise to develop the evidence base but, critically, also in our efforts to translate that evidence into meaningful, accessible, and impactful messages; programs; policies; and standards of care in the United States and worldwide.

## Funding

The BEGIN Project was initiated by the Pediatric Growth and Nutrition Branch of the *Eunice Kennedy Shriver* National Institute of Child Health and Human Development (NICHD) of the National Institutes of Health (NIH) in partnership with the Bill & Melinda Gates Foundation (BMGF) and the Academy of Nutrition and Dietetics (Academy). The publication of this supplement was made possible by the NICHD, and support for assistance (by BioCentric, Inc.) with editing, proofing, and submitting the manuscripts was also provided by the NICHD.
