# Supplementary material for: The “Breastmilk Ecology: Genesis of Infant Nutrition (BEGIN)” Project – executive summary
Source: Am J Clin Nutr. 2023 May 10;117(Suppl 1):S1–S10. doi: 10.1016/j.ajcnut.2022.12.020 (PMC10356555; doi:10.1016/j.ajcnut.2022.12.020)
Supplement: Multimedia component 1 [file mmc1.docx]

**Supplemental File for**

**The “Breastmilk Ecology: Genesis of Infant Nutrition (BEGIN)”
project – executive summary**

Daniel J. Raiten, Alison L. Steiber, Constantina Papoutsakis, Mary Rozga, Deepa Handu, Gabriela V. Proaño, Lisa Moloney, Andrew A. Bremer

**Supplemental Table 1**

Scientific Steering Committee (SSC)

Roster of Members

Jennifer M. Nelson, MD, MPH, FAAP

LCDR U.S. Public Health Service

Nutrition Branch

Division of Nutrition, Physical Activity, Obesity

National Center for Chronic Disease Prevention and Health Promotion

US Centers for Disease Control and Prevention

Kotaro Kaneko, PhD

Staff Scientist

Center for Food Safety

US Food and Drug Administration

Andrea Lotze, MD

Medical Director, Infant Formula and Medical Foods Staff

Center for Food Safety

US Food and Drug Administration

Steve Abrams, MD

Professor, Department of Pediatrics

The University of Texas at Austin

Sun-Eun (Sunny) Lee, PhD

Program Officer

Bill & Melinda Gates Foundation

Alison Steiber, PhD, RD

Chief Science Officer

Academy of Nutrition and Dietetics

Lindsay Allen, PhD

Research Scientist

Western Human Nutrition Research Center

Agriculture Research Service

US Department of Agriculture

Valery Soto, MS, RD, CLC

Senior Nutritionist

Nutrition Services Branch

Supplemental Nutrition and Safety Programs

Supplemental Foods Program Division

US Department of Agriculture Food and Nutrition Service

**Supplemental Table 2: WG Rosters**

***WG 1: Parental Factors***

Margaret C. (Peggy) Neville, PhD (**CHAIR**) Professor Emerita

Department of Physiology and Biophysics University of Colorado School of Medicine

Mark McGuire, PhD

University Distinguished Professor

Director of the Idaho Agricultural Experiment Station

Associate Dean

College of Agricultural and Life Sciences University of Idaho

Ellen Demerath, PhD

Professor, Division of Epidemiology and Community Health

Affiliate Faculty, Center for Neurobehavioral Development

Member, Obesity Prevention Center University of Minnesota

Jennifer Hahn-Holbrook, PhD Assistant Professor of Psychology Director of the UC Merced Latch Lab UC- Merced

Kathleen Rasmussen, PhD

The Nancy Schlegel Meinig Professor of Maternal and Child Nutrition

Division of Nutritional Sciences Cornell University

Michael C. Rudolph, PhD Assistant Professor of Physiology

Choctaw Nation Chair in Adult Endocrinology Harold Hamm Diabetes Center

The University of Oklahoma Health Sci. Center

Russ Hovey, PhD

Professor, Department of Animal Science University of California, Davis

Edward Newton Professor of Ob/Gyn Brody School of Medicine East Carolina University

**Academy of Nutrition and Dietetics:**

Constantina (Tina) Papoutsakis, PhD, RDN

***WG 2: Milk Composition***

Michelle (Shelley) McGuire, PhD (**CHAIR**) Professor and Director

Maternal and Child Nutrition

College of Agricultural and Life Sciences Margaret Ritchie School of Family and Consumer Sciences

University of Idaho

Lindsay H. Allen, PhD
Western Human Nutrition Research Center

Agriculture Research Service

US Department of Agriculture

Adjunct Research Professor University of California, Davis

David Sela. PhD

Assistant Professor, Dept. of Food Science Adjunct Professor, Dept. of Microbiology Faculty, Organismic and Evolutionary Biology Faculty, Molecular and Cellular Biology

U Mass-Amherst

Dave Dallas, PhD Assistant Professor Department of Nutrition

School of Biological and Population Health Sciences

Oregon State University

Jennifer Smilowitz. PhD

Associate Director of Human Studies Research Program Foods for Health Institute University of California, Davis

James McManaman, PhD

Professor-Director, Graduate Program in Integrative Physiology

Division of Reproductive Sciences Department of Obstetrics and Gynecology University of Colorado

Antti Seppo, PhD

Research Associate Professor Department of Pediatrics

Pediatric Allergy/Immunology (SMD) University of Rochester

Janet Williams

Senior Research Scientist

Department of Animal, Veterinary & Food Sciences

University of Idaho

Bridget E. Young, PhD, CLC Research Assistant Professor

University of Rochester School of Medicine and Dentistry

Department of Pediatrics; Allergy and Immunology

University of Rochester

**Academy of Nutrition and Dietetics:**

Mary Rozga, PhD, RDN

***WG 3: Infant Factors***

Nancy Krebs, MD, MS (**CHAIR**) Professor, Pediatrics-Nutrition Head, Section of Nutrition Vice Chair, Academic Affairs Department of Pediatrics University of Colorado

Julie Mennella, PhD
Developmental Psychobiologist

Monell Chemical Senses Center

Mandy Belfort, MD

Assistant Professor of Pediatrics, Harvard Medical School

Paula Meier, PhD, RN

Professor of Pediatrics and Nursing

Rush University Medical Center

Deborah L. O’Connor, PhD, RD

Earle W. McHenry Professor, and Chair, Department of Nutritional Sciences Faculty of Medicine, University of Toronto Scientist, The Hospital for Sick Children

Sarah N. Taylor, MD, MSCR

Director of Clinical Research, Division of Neonatology

Associate Professor, Department of Pediatrics Yale School of Medicine

**Academy of Nutrition and Dietetics:**

Deepa Handu, PhD, RDN

***WG 4: Integration and Application***

Sharon Donovan, PhD, RD (**CHAIR**)

Professor &Melissa M. Noel Endowed Chair in Nutrition and Health

Department of Food Science & Human Nutrition University of Illinois, Urbana

Nima Aghaeepour, PhD Assistant Professor (Research) of

Anesthesiology, Perioperative, & Pain Medicine Baxter Laboratory in Stem Cell Biology, Stanford University

Aline Andres, PhD

Associate Director/Professor Department of Pediatrics

Section of Developmental Nutrition

University of Arkansas School of Medicine and Arkansas Children’s Nutrition Center

Meghan Azad, PhD

Canada Research Chair, Developmental Origins of Chronic Disease

Associate Professor, Pediatrics and Child Health, University of Manitoba

Susan Carlson, PhD Associate Dean for Research

Program Director, Doctorate in Medical Nutrition Science

AJ Rice Professor of Nutrition Department of Dietetics and Nutrition University of Kansas, Kansas City

Kirsi Jarvinen-Seppo. MD, PhD

Associate Professor of Pediatrics, Medicine, Microbiology and Immunology

Chief and Founders’ Distinguished Chair in Pediatric Allergy and Immunology

Director, Center for Food Allergy, FARE Clinical Network

University of Rochester School of Medicine/ Golisano Children's Hospital

Weili Lin, PhD

Director, Biomedical Research Imaging Center Dixie Lee Boney Soo Distinguished Professor of Neurological Medicine

Professor and Vice Chair of Basic Research, Radiology

Professor, Biomedical Engineering, Neurology and School of Pharmacy

University of North Carolina, Chapel Hill

Bo Lonnerdal, PhD

Distinguished Professor Emeritus Department of Nutrition and Department of Internal Medicine University of California, Davis

Carolyn Slupsky, PhD Professor

Department of Nutrition and Department of Food Science & Technology

Chair, Graduate Group in Nutritional Biology Kinsella Endowed Chair in Food, Nutrition, and Health

Nutritionist in Agricultural Experiment Station University of California, Davis

**Academy of Nutrition and Dietetics:**

Alison Steiber PhD, RDN

***WG 5: Translation and Implementation***

Laurie Nommsen-Rivers, PhD, RD, IBCLC (**CHAIR**)
Associate Professor of Nutrition

Ruth Rosvear Endowed Chair of Maternal and Child Nutrition

University of Cincinnati

Maureen Black, PhD

John A. Scholl, MD and Mary Louise Scholl, MD Professor in Pediatrics

University of Maryland School of Medicine

Michal A Young, MD Neonatologist

Associate Professor and Director of NICU and Newborn Nurseries

Department of Pediatrics and Child Health Howard University Hospital and Howard University College of Medicine

Kiersten Israel-Ballard, DrPH

Associate Director - Maternal, Newborn and Child Health/Nutrition

PATH

Meredith Jane Heinig, PhD Professor, Department of Nutrition

Director & International Board Certified Lactation Consultant, Human Lactation Center University of California, Davis

Sharon Groh-Wargo, PhD, RDN Professor of Pediatrics and Nutrition

Case Western Reserve University School of Medicine

Neonatal Nutritionist Department of Pediatrics MetroHealth Medical Center

Alison Stuebe, MD, MSc

Professor, Maternal-Fetal Medicine

Interim Director of the Division of Maternal- Fetal Medicine

Medical Director, Lactation Services

Co-Director UNC Center for Maternal and Infant Health

Distinguished Scholar in Infant and Young Child Feeding, UNC Gillings School of Global Public UNC-Chapel Hill

Parul Christian, PhD

Director of Program in Human Nutrition Professor

International Health (Primary) Division: Human Nutrition Center for Global Health Center for Human Nutrition Johns Hopkins University

Julie Obbagy, PhD, RD Nutritionist

Nutrition Evidence Systematic Review, Office of Nutrition Guidance and Analysis.

Center for Nutrition Policy and Promotion, Food and Nutrition Service

U.S. Department of Agriculture

**Academy of Nutrition and Dietetics:**
Gabriela Proano, MS, RDN

Lisa Moloney, MS, RDN
